# IL-18, but not IL-15, contributes to the IL-12-dependent induction of NK-cell effector functions by *Leishmania infantum in vivo*

**DOI:** 10.1002/eji.200939988

**Published:** 2010-03-08

**Authors:** Simone Haeberlein, Heidi Sebald, Christian Bogdan, Ulrike Schleicher

**Affiliations:** Microbiology Institute – Clinical Microbiology, Immunology and Hygiene, Friedrich-Alexander-University Erlangen-Nuremberg and University Clinic of ErlangenErlangen, Germany

**Keywords:** IL-12, IL-15, IL-18, Leishmaniasis, NK cell

## Abstract

Activation of NK cells is a hallmark of infections with intracellular pathogens. We previously showed that the protozoan parasite *Leishmania infantum* triggered a rapid NK-cell response in mice that required TLR9-positive myeloid DC and IL-12, but no IFN-α/β. Here, we investigated whether IL-15 or IL-18 mediate the activity of IL-12 or function as independent activators of NK cells. In contrast to earlier studies that described IL-15 as crucial for NK-cell priming in response to TLR ligands, the expression of IFN-γ, FasL, perforin and granzyme B by NK cells in *L. infantum*-infected mice was completely preserved in the absence of IL-15, whereas the proliferative capacity of NK cells was lower than in WT mice. IFN-γ secretion, cytotoxicity and FasL expression of NK cells from infected IL-18^−/−^ mice were significantly reduced compared with controls, but, unlike IL-12, IL-18 was not essential for NK-cell effector functions. Part of the NK-cell-stimulatory effect of IL-12 was dependent on IL-18. We conclude that IL-15 is not functioning as a universal NK-cell priming signal and that IL-18 contributes to the NK-cell response in visceral leishmaniasis. The cytokine requirements for NK-cell activation appear to differ contingent upon the infectious pathogen.

## Introduction

NK cells are important effector cells of the innate immune response to infectious pathogens and malignant cells. They contribute to the control of infections and tumors by producing cytokines such as IFN-γ, lysing transformed or virally infected target cells, and by supporting the development of a protective T-cell response 1–4. Later during infection NK cells might also acquire an immunoregulatory activity 5.

An NK-cell response is readily induced during infections with intracellular pathogens and requires cytokine and receptor signals that are delivered by myeloid cells 6–8. Recently, myeloid DC (mDC) and neutrophils have been shown to prime NK cells during viral, bacterial or protozoan infections *in vivo* 9–11. Notably, various cytokines of myeloid cell origin, such as IFN-α/β 9, IL-12p70 10, IL-15 9 and IL-18 11, were identified to account for the activation of mature NK cells. This suggests that different pathogens might trigger distinct NK-cell activation pathways, or, alternatively, that the production or function of the NK-cell-activating cytokines is interdependent so that the respective gene-deficient mice exhibit a similar defect of NK-cell activation. In the case of IL-12 and IL-18, not only direct and independent, but also cooperative effects on NK cells have been described 11–13. A particular convincing example for the sequential action of two cytokines was observed after activation of NK cells by ligands of TLR3, TLR4, TLR7 or TLR9, which was strictly dependent on IFN-α/β signaling as well as IL-15. mDC from WT, but not from IL-15^−/−^ or IFN-α/β-receptor^−/−^ mice were able to prime NK cells *in vitro* 14 and *in vivo* 9. As TLR-induced IFN-α/β triggered the production of IL-15 9,14,15, the IFN-α/β-dependent NK-cell activation is thought to occur *via* the release of endogenous IL-15, which binds to the IL-15Rα chain on the surface of the mDC producer cell and is then transpresented to IL-15-responsive NK cells expressing the IL-15Rβ chain and the IL-2R common γ chain 16,17. As IL-15 also cooperates with IL-18 18, induces IL-12 by myeloid cells 19,20, and is essential for NK-cell expansion and activation during viral, bacterial and fungal infections 9,16,21–26, it can be considered as a central cytokine not only for normal NK-cell homeostasis 17,27,28 but also for an active NK-cell response.

When mice are cutaneously or intravenously infected with protozoan parasites of the species *Leishmania (L.) major* or *L. infantum*, which cause cutaneous or visceral leishmaniasis, NK cells become rapidly activated in the draining lymph node or spleen, respectively, and contribute to parasite control (reviewed in 29). We previously showed that the activation of NK cells in these models required mDC, sensing of the parasites by TLR9 and the release of IL-12, but was independent of IFN-α/β despite eliciting a strong IFN-α/β response in plasmacytoid DC 10,30. These studies, however, did not exclude the contribution of additional cytokine signals acting independently or up- or downstream of IL-12. In the present report we therefore addressed three questions using the mouse model of experimental visceral leishmaniasis: (i) Does IL-15 function as NK-cell-stimulatory cytokine as predicted from the results obtained in other systems? (ii) Is IL-18 similar to IL-12 a prerequisite for NK-cell activation in visceral leishmaniasis? (iii) Do IL-15 and IL-18 operate independently of IL-12? Our data clearly illustrate that the full induction of NK-cell effector functions in visceral leishmaniasis requires IL-12 and IL-18, but – unexpectedly – is entirely independent of IL-15. Whereas IL-12 is essential for the expression of NK-cell effector functions *in vivo*, IL-18 only plays a contributory role by supporting the NK-cell response to IL-12.

## Results

### Induction of NK-cell effector functions by *L. infantum* is independent of IL-15

Considering the known mode of action of IL-15 *via* transpresentation 16,17, we first tested the expression of the IL-15Rα-chain on splenic mDC. At 24 h after infection with *L. infantum,* we observed a 2- to 2.5-fold increase of the percentage of IL-15Rα^+^ mDC in the spleen, indicating that *L. infantum* might modulate the IL-15/IL-15R system (Supporting Information Fig. 1).

**Figure 1:**
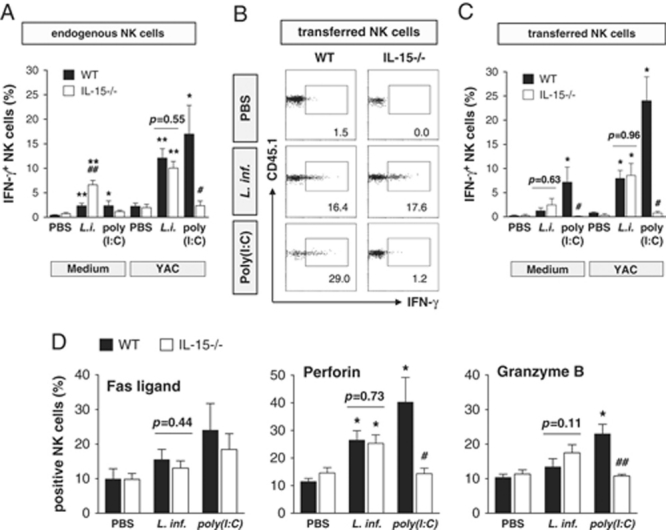
IL-15 is dispensable for NK-cell activation in *L. infantum*-infected mice. C57BL/6 WT or IL-15^−/−^ mice were injected i.v. with 1×10^7^ *L. infantum* promastigote parasites or PBS or i.p. with 50 μg poly(I:C). (A) Intracellular IFN-γ protein expression in endogenous NK cells taken from WT and IL-15^−/−^ mice at 12 h after infection and cultured for 4 h *in vitro* in medium alone or in the presence of YAC-1 cells. Mean±SEM of four independent experiments with two to three mice *per* group. (B and C) Intracellular IFN-γ protein expression of donor NK cells after transfer into WT and IL-15^−/−^ mice. Adoptive transfer of CD3^−^NK1.1^+^CD45.1^+^ NK cells from naïve WT donor mice was performed 2–4 h after *L. infantum* infection, PBS or poly(I:C) treatment of CD45.2^+^ WT or IL-15^−/−^ recipients. Twelve hours later splenocytes were prepared, cultured in medium or in the presence of YAC-1 cells for 4 h, and stained for CD3^−^NK1.1^+^CD45.1^+^ NK cells and intracellular IFN-γ. (B) Dot plot analysis of one representative of three experiments with two to three mice *per* group after *in vitro* restimulation with YAC-1 cells is given. (C) Mean±SEM of three independent experiments with two to three mice *per* group. (D) Surface expression of FasL and intracellular expression of perforin or gzmB of endogenous CD3^−^NK1.1^+^ NK cells from WT and IL-15^−/−^ directly *ex vivo*. Mean±SEM of three to four experiments with two to three mice *per* group. (A–D) Significant differences by Mann–Whitney test between cells from infected, poly(I:C) or IL-15-treated and PBS-treated mice (^*^p<0.05; ^**^p<0.002) and between WT and IL-15^−/−^ (^♯^*p*<0.05; ^♯♯^*p*<0.005) are indicated.

**Figure 2:**
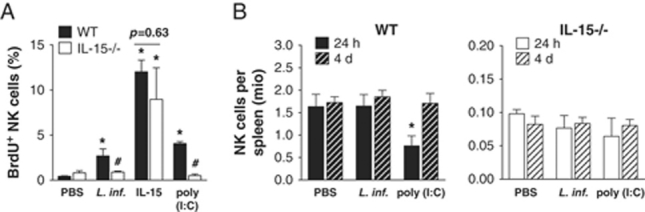
Infection with *L. infantum* causes an IL-15-dependent NK-cell proliferation, which does not augment the splenic NK-cell compartment. 1×10^7^ *L. infantum* promastigote parasites or PBS were injected i.v. in C57BL/6 WT or IL-15^−/−^ mice. (A) NK-cell proliferation in WT and IL-15^−/−^ mice 24 h upon infection, poly(I:C) or IL-15/sIL-15Rα treatment measured by BrdU incorporation. Twenty-two hours p.i. 1 mg BrdU *per* mouse was injected i.p. and 2 h later the percentage of proliferated BrdU^+^ NK cells was analysed by FACS. Mean±SEM of two independent experiments with two mice *per* group. Significant differences by Mann–Whitney test between cells from infected, poly(I:C) or IL-15-treated and PBS treated mice (^*^p<0.05; ^**^p<0.002) and between WT and IL-15^−/−^ (^♯^*p*<0.05; ^♯♯^*p*<0.005) are indicated. (B) Absolute NK1.1^+^CD3^−^ NK-cell numbers in the spleen were determined by FACS analysis at days 1 and 4 of infection. Mean±SEM of three independent experiments with two to three mice *per* group is given. Significant differences by Mann–Whitney test between cells from poly(I:C) and PBS treated mice (^*^p<0.05) are indicated.

Next, we analysed whether IL-15 is required for the induction of NK-cell effector functions *in vivo*. As IL-15 is critical for NK-cell development and maturation 21,31, the spleen of IL-15^−/−^ mice contained a dramatically reduced percentage and absolute number of mature NK cells compared with WT mice (0.16±0.03% *versus* 2.8±0.9% NK1.1^+^CD3^−^ cells; mean±SD of seven or ten experiments, respectively; see also PBS-treated groups of Fig. 2B). We first investigated the *L. infantum*-induced expression of IFN-γ 12 h post infection (p.i.) within the residual endogenous NK1.1^+^CD3^−^ NK-cell population of IL-15^−/−^ mice as compared with NK cells of WT controls after a 4 h culture period, either in medium alone or in the presence of YAC-1 target cells. As expected, the *in vitro* restimulation augmented the IFN-γ release of *in vivo* primed NK cells (Fig. 1A). Irrespective of the culture condition we did not observe a significant reduction of the percentage of IFN-γ^+^ NK cells in *L. infantum*-infected mice in the absence of IL-15; in fact, without YAC cell restimulation the IFN-γ production by NK cells from infected IL-15^−/−^ mice was even significantly higher than by NK cells from WT controls (Fig. 1A, medium). In contrast, the TLR3-dependent activation of NK cells elicited by poly(I:C) was drastically reduced in IL-15^−/−^ mice confirming previously reported results 9. Thus, IL-15 was clearly dispensable for the *L. infantum*-induced expression of IFN-γ by NK cells *in vivo*.

It was necessary to exclude the possibility that the residual NK-cell population in IL-15^−/−^ mice had acquired IL-15-independent, alternative modes of activation and therefore did not exhibit the same stimulation requirements as NK cells in WT mice. To this end we analysed the *L. infantum*-induced expression of IFN-γ in highly purified NK cells from uninfected congenic CD45.1^+^ WT donor mice that had been transferred into *L. infantum*-infected WT or IL-15^−/−^ recipient mice 2 to 4 h after infection (Fig. 1B and C). As the NK-cell activation was determined 12 h after cell transfer, the duration of the experiment was short enough to allow for the survival of transferred NK cells in the IL-15-negative microenvironment. Independent of the *in vitro* culture conditions the *L. infantum*-induced expression of IFN-γ in transferred CD45.1^+^ NK cells remained unaffected by the absence of IL-15 in the recipients. Neither the percentage (Fig. 1B and C) nor the MFI of IFN-γ^+^ donor NK cells (WT: 58.20±0.49, IL-15^−/−^: 53.89±5.78 (culture in medium); WT: 82.26±14.32, IL-15^−/−^: 74.59±16.46 (YAC-1 restimulation); mean±SEM of three experiments) or the absolute number of IFN-γ^+^ donor NK cells (WT: 651±99, IL-15^−/−^: 1641±324 (culture in medium); WT: 4733±744, IL-15^−/−^: 5444±1211 (YAC-1 restimulation); mean±SEM of three experiments) was significantly lower in infected IL-15^−/−^ recipient mice compared with WT controls. In contrast, the TLR3-dependent activation of NK cells elicited by poly(I:C) was entirely dependent on IL-15 (Fig. 1B and C).

To test whether IL-15 is required for the induction of NK-cell cytotoxicity *in vivo*, we analysed the expression of critical components of the cytotoxic machinery of NK cells (Fas-FasL intercellular linkage-mediated pathway; granule-dependent exocytosis pathway), because the very low number of differentiated NK cells in IL-15^−/−^ mice stymies the direct measurement of NK-cell cytotoxicity. *L. infantum* caused a modest upregulation of the percentages of FasL^+^, perforin^+^ and granzyme (gzm) B^+^ NK cells in the spleen, which was indistinguishable in WT and IL-15^−/−^ mice, whereas the induction of perforin and gzmB in response to poly(I:C) was clearly impaired in NK cells from IL-15^−/−^ mice (Fig. 1D). Thus, the induction of both NK-cell effector functions (IFN-γ release and cytotoxicity) by *L. infantum* is completely preserved in the absence of IL-15.

### IL-15-dependent proliferation of NK cells in *L. infantum*-infected mice

Since signals through the IL-2Rγ/IL-15Rβ on NK cells are also known to stimulate NK-cell proliferation and accumulation 17,22,24,32,33, we tested the NK-cell expansion in *L. infantum*-infected WT *versus* IL-15^−/−^ mice 24 h p.i. by BrdU incorporation. As a positive control both mouse groups were treated i.p. with pre-complexed IL-15 and sIL-15R-Fc, a “superagonist” of NK-cell proliferation 34. The injection of the parasites triggered a small, but significant NK-cell proliferation in the spleen of WT mice during the first day of infection, which was not observed in the absence of IL-15 (Fig. 2A). The *L. infantum*-induced and IL-15-dependent NK-cell proliferation, however, did not lead to an expansion of the splenic NK-cell compartment. During the early phase of infection (day 1 until day 4) the absolute number of NK cells *per* spleen remained unaltered in both WT and IL-15^−/−^ mice compared with the respective uninfected control mice (Fig. 2B). In contrast, poly(I:C) treatment caused an IL-15-dependent decrease of the splenic NK-cell number, which presumably reflects the migration of primed and activated NK cells to the periphery 9.

### The activation of NK cells in IL-15^−/−^ mice requires IL-12

**Figure 3:**
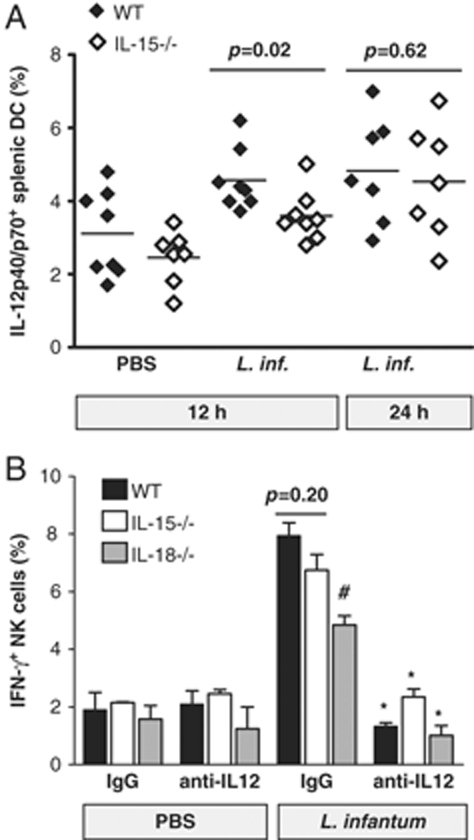
Activation of NK cells in IL-15^−/−^ mice strictly depends on IL-12. C57BL/6 WT, IL-15^−/−^ or IL-18^−/−^ mice were injected i.v. with 1×10^7^ *L. infantum* promastigote parasites or PBS. (A) Intracellular IL-12 p40/p70 expression in splenic mDC (CD11b^int^CD11c^hi^ cells) taken from WT and IL-15^−/−^ mice at 12 or 24 h after infection and cultured *in vitro* for 4 h in medium. Three independent experiments with two to three mice *per* group. Each diamond represents a single mouse; the mean is given as horizontal line. (B) Intracellular IFN-γ protein expression in NK cells taken from WT, IL-15^−/−^ or IL-18^−/−^ mice 12 h after infection and co-cultured for 4 h *in vitro* with YAC-1 cells; 30 min before injection of parasites or PBS IL-12 p40-neutralizing mAb or rat IgG control Ab were applied i.p. Mean±SEM of two independent experiments with two mice *per* group. Significant differences by Mann–Whitney test between anti-IL-12p40 and control IgG treatment are indicated as ^*^p<0.05; between WT and IL-15^−/−^ or IL-18^−/−^ NK cells as ^♯^*p*<0.05.

**Figure 4:**
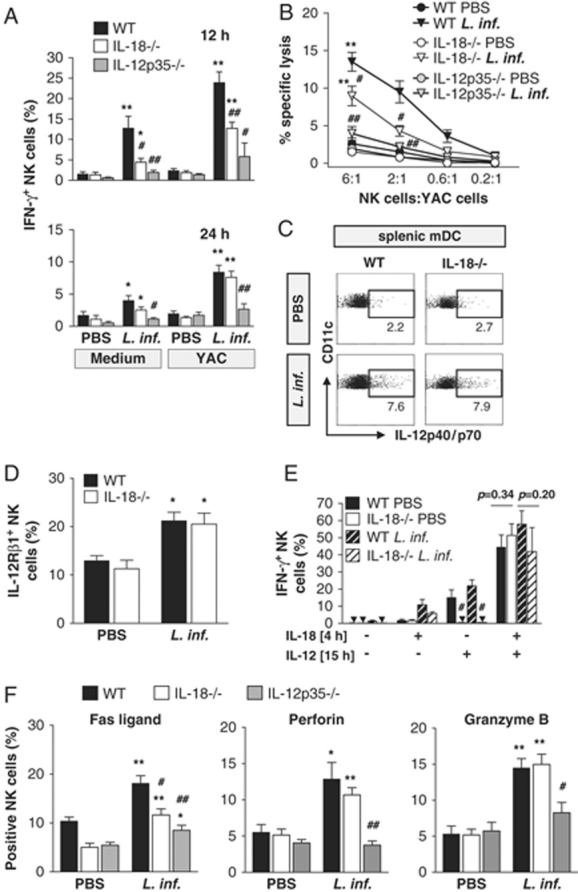
Mechanism of NK-cell activation by IL-18 in *L. infantum*-infected mice. At 12 and 24 h after i.v. injection of PBS or *L. infantum* promastigotes into C57BL/6 WT, IL-18^−/−^ and IL-12p35^−/−^ mice, splenocytes were prepared. (A) Frequency of IFN-γ^+^ NK cells (CD3^−^NK1.1^+^) as determined by intracellular cytokine staining and FACS analysis after a 4 h culture in medium or with YAC cells. Mean±SEM of six (WT *versus* IL-18^−/−^) and three (WT *versus* IL-18^−/−^ and IL-12p35^−/−^) independent experiments with two to three mice *per* group. (B) NK-cell cytotoxicity at 24 h after infection. Mean±SEM of four (WT *versus* IL-18^−/−^) and three (WT *versus* IL-18^−/−^ and IL-12p35^−/−^) independent experiments with two to three mice *per* group. (C) Intracellular IL-12p40/p70 protein expression of splenic mDC (restimulated in culture medium for 4 h; gated on CD11b^int^CD11c^hi^ cells) is shown in a dot plot analysis representative for three independent experiments with two to three mice *per* group. (D) Surface expression of IL-12Rβ1 on CD3^-^NK1.1^+^ NK cells directly *ex vivo*. Mean±SEM of three experiments with two to three mice *per* group. (E) Intracellular IFN-γ protein expression in NK cells from WT and IL-18^−/−^ mice 12 h after infection or PBS treatment, which were cultured for 4 h (±IL-18), washed, and further incubated for 15 h (±IL-12). Mean±SEM of three independent experiments with two mice *per* group. (F) Surface expression of FasL and intracellular expression of perforin or gzmB of CD3^-^NK1.1^+^ NK cells directly *ex vivo*. FasL: mean±SEM of five (WT *versus* IL-18^−/−^) and three (WT *versus* IL-18^−/−^ and IL-12p35^−/−^) experiments, two to three mice *per* group; perforin: mean±SEM of three experiments, two mice *per* group; gzmB: mean±SEM of four (WT *versus* IL-18^−/−^) and three (WT *versus* IL-18^−/−^ and IL-12p35^−/−^) experiments, two to three mice *per* group. (A–F) Significant differences by Mann–Whitney test between cells from infected and PBS treated mice (^*^p<0.05; ^**^p<0.002) and between WT and IL-18^−/−^ or IL-12p35^−/−^ (^♯^*p*<0.05; ^♯♯^*p*<0.005) are indicated.

Having seen that IL-15 is dispensable for induction of NK-cell effector functions by *L. infantum*, we addressed the question, whether the *L. infantum*-induced NK-cell response in IL-15^−/−^ mice is still strictly dependent on IL-12 as seen in WT mice 10 (see also below in text and Fig. 4). We first checked the secretion of IL-12 by splenic mDC of WT and IL-15^−/−^ mice following infection (Fig. 3A). In both mouse groups IL-12 was induced upon infection, although mDC-derived IL-12p40/p70 was slightly reduced in IL-15-deficient mice at the 12 h time point. The fact that this difference was no longer detectable at 24 h p.i. (Fig. 3A) argues against a profound and biologically relevant difference in the production of IL-12 by mDC of both mouse strains. This view is strongly supported by the fact that in all our NK-cell transfer experiments (see Fig. 1C) the percentage of IL-12p40/p70^+^ splenic mDC (∼4–5%) 14–16 h p.i. was comparable in infected WT and IL-15^−/−^ mice (data not shown). Furthermore, neutralization of IL-12 by Ab-treatment prior to infection completely prevented NK cell IFN-γ production in both WT and IL-15^−/−^ mice (Fig. 3B). Anti-IL-12 had exactly the same effect, when the NK cell IFN-γ expression was determined without *in vitro* restimulation, except that the percentages of IFN-γ^+^ NK cells were lower in all groups (data not shown). Thus, even under IL-15-deficient conditions IL-12 is the key cytokine for *L. infantum*-induced NK-cell activation.

### Endogenous IL-18 contributes to the activation of NK cells in visceral leishmaniasis

As IL-18 is known to activate mouse NK cells *in vitro* and *in vivo* 12,35, we tested whether IL-18 contributes to the NK-cell activation in visceral leishmaniasis. At the height of innate IFN-γ production in the spleen (12 h p.i.) the percentage of IFN-γ protein-positive NK cells from IL-18^−/−^ mice was reduced by roughly 50% compared with WT mice (with or without *in vitro* restimulation) (Fig. 4A). Notably, infection with *L. infantum* did lead to an upregulation of IFN-γ mRNA in partially (70%) or highly purified splenic NK cells (>99.9%) and the levels of IFN-γ mRNA were comparable in WT and IL-18^−/−^ mice (Supporting Information Fig. 2; data not shown). The cytotoxic activity of NK cells from IL-18^−/−^ mice was significantly lower compared with WT NK cells (Fig. 4B). As seen before 10 the IFN-γ production as well as the cytotoxic activity of NK cells in *L. infantum*-infected IL-12^−/−^ mice was nearly completely blocked (Fig. 4A and B). Thus, NK-cell activation is partially maintained in IL-18^−/−^ mice, but abolished in IL-12^−/−^ mice.

### Functional relationship between IL-12 and IL-18 during activation of NK cells in visceral leishmaniasis

Earlier studies, which used naïve, non-infected mice, proposed that IL-18 either acts on NK cells in an IL-12-independent manner or that it increases the IL-12 responsiveness of NK cells 12,13. We therefore performed experiments to define how IL-18 might promote the IFN-γ production and the cytotoxicity of NK cells in visceral leishmaniasis. First, we searched for a possible impact of IL-18 on the expression of IL-12, which is essential for NK-cell activation in *L. infantum*-infected mice 10. We obtained no evidence that IL-18 regulates the induction of IL-12 by *L. infantum* in splenic DC *in vivo* (Fig. 4C) or in BM-derived DC or macrophages *in vitro* (Supporting Information Fig. 3), thus excluding the possibility that IL-18 functions *via* increasing the availability of IL-12. Second, we observed that the surface expression of the IL-12Rβ1-chain and the mRNA expression of IL-12Rβ2-chain of NK cells from WT and IL-18^−/−^ mice were comparable (Fig. 4D and Supporting Information Fig. 4), which argues for an IL-12R-independent effect of IL-18. Third, we tested whether IL-18 might prime NK cells from *L. infantum*-infected mice for a response to IL-12 as previously reported for naïve mice 13. For this purpose spleen cells were harvested from PBS-treated or *L. infantum*-infected WT or IL-18^−/−^ mice 12 h p.i. and cultured in the presence or absence of IL-18 for 4 h, followed by a 15 h incubation with or without IL-12. Thereafter, the IFN-γ production of NK cells was measured. Confirming earlier results 13, the *in vitro* induction of IFN-γ in naïve NK cells by IL-12 required prior *in vivo* priming by IL-18. In NK cells from IL-18^−/−^ mice the IFN-γ expression could be restored by *in vitro* pre-stimulation with IL-18, which further augmented the IFN-γ release by NK cells of both mouse strains. Identical results were obtained with NK cells from infected mice (Fig. 4E). These *ex vivo* data suggest that priming by IL-18 is required to achieve full IL-12-mediated NK-cell activity during the early phase of *L. infantum* infection. However, it is important to emphasize that the induction of NK cell IFN-γ secretion and NK-cell cytotoxicity *in vivo* were only partially blocked in IL-18^−/−^ mice, whereas both NK-cell functions were almost completely abolished in the absence of IL-12 (Fig. 4A and B). In addition, the production of IFN-γ by NK cells from IL-18^−/−^ mice was eliminated when IL-12 was neutralized (Fig. 3B). Finally, we investigated whether IL-18 regulates FasL, perforin and/or gzmB expression. Although *L. infantum* infection caused an increase of the percentages of FasL^+^ NK cells in the spleen of both WT and IL-18^−/−^ mice, the numbers of FasL^+^ NK cells were significantly lower in the case of IL-18^−/−^ mice (Fig. 4F). In contrast, a deficiency of IL-18 did not affect the expression of gzmB or perforin mRNA and protein in NK cells (Fig. 4F and Supporting Information Fig. 5). In IL-12^−/−^ mice none of these cytotoxic effector molecules was induced upon infection (Fig. 4F).

Together, these data support the notion that IL-18 contributes to the IL-12-mediated NK cell IFN-γ response by increasing the IL-12 responsiveness of NK cells. In addition, the results clearly illustrate that IL-12 also exerts IL-18-independent NK-cell-stimulatory effects.

## Discussion

We previously reported an essential role of mDC-derived IL-12 for NK-cell activation in visceral leishmaniasis, whereas IFN-α/β was largely dispensable 10. In the present study we provide novel insights into the cytokine signals and mechanisms that underlie the process of NK-cell activation during *L. infantum* infection. First, our results demonstrate that the IL-12-dependent stimulation of NK cell IFN-γ production and cytotoxicity did not require the activity of IL-15 as shown by the analysis of residual NK cells in IL-15^−/−^ mice or the transfer of WT NK cells into *L. infantum*-infected IL-15^−/−^ mice. Only the modest enhancement of NK-cell proliferation in *L. infantum*-infected mice was mediated by IL-15. Second, even in the strict absence of IL-15 no compensatory IL-12-independent mechanism for the activation of NK-cell effector functions emerged. Third, IL-18 helped to trigger NK-cell effector functions in *L. infantum* infection, but comparing the NK-cell response in WT, IL-12^−/−^ and IL-18^−/−^ mice it became readily apparent that IL-18 played a contributory, but not an essential role. Finally, *in vivo* and *ex vivo* experiments demonstrated that IL-18 facilitated the response of NK cells to IL-12, but also proved that IL-12 is capable to stimulate NK cells in the absence of IL-18. There was clearly a differential regulation of FasL, perforin and gzmB in IL-18^−/−^ *versus* IL-12^−/−^ mice.

The IL-18-independent effect of IL-12 on NK-cell activation in visceral leishmaniasis might result from an increased nuclear translocation of STAT4 and/or NF-κB as previously seen in NK cells present in unseparated mouse macrophage populations 36,37. The enhancement of NK-cell cytotoxicity by IL-18 in *L. infantum*-infected mice is most likely due to the observed increased FasL-expression, which was shown to account for the antitumor effects of a treatment with IL-18 38,39.

Our results contrast with earlier findings obtained in other infection models using viral, bacterial or fungal pathogens, where IFN-α/β and/or IL-15 enhanced the activation of NK cells or were critical for NK cell IFN-γ production and/or for NK-cell cytotoxicity 1,9,22,25,26. With respect to the regulation of the expression of NK-cell cytotoxic granule proteins Fehniger *et al*. identified IL-2 and IL-15 as cytokines that were most potent in switching on the translation of perforin and gzmB in mouse NK cells *in vitro*, whereas IL-12 showed only a mild effect 40. These data differ markedly from our *in vivo* observations in visceral leishmaniasis, where we noted a strictly IL-12-dependent, but IL-15-independent increase of perforin and gzmB protein in NK cells (Figs. 1D and 4F). In accordance with earlier *in vitro* studies 40 our data provide evidence that perforin and gzmB are regulated by a post-transcriptional mechanism also *in vivo*, because up-regulation of both molecules was only visible on the protein level, but not on the mRNA level (Fig. 4F and Supporting Information Fig. 5).

There is evidence that an IL-15-independent activation of NK cells might also occur during other protozoan infections. Although the necessary *in vivo* experiments have not yet been reported, at least *in vitro* splenic DC from *Plasmodium chabaudi*-infected IL-15^−/−^ mice were as potent as DC from WT mice to activate partially purified NK cells for the expression of IFN-γ 41. Even during certain viral infections IL-15 might be dispensable for NK-cell activation. During the preparation of our manuscript Sun *et al*. published that an infection with mouse CMV (MCMV) elicits a massive expansion of NK cells (72-fold within 7 days) in IL-15Rα-deficient mice and that these NK cells can be activated *in vitro* for the expression of IFN-γ and target cell cytotoxicity 42. As shown in the present study, *L. infantum* also caused a proliferation of NK cells (Fig. 2A), but unlike to the MCMV model it required IL-15 and did not lead to a measurable expansion and accumulation of NK cells in the spleen (Fig. 2B). This is most likely due to several circumstances. First, the NK-cell proliferation induced by *L. infantum* was quite weak. Only approximately 2.5% of all NK cells incorporated BrdU during the final 2 h of a 24 h infection period, whereas in the MCMV model a vigorous NK-cell proliferation with 21% BrdU^+^ NK cells was reported at day 1.5 of infection 43. Second, the modest NK-cell proliferation conveyed by IL-15 might reflect the less than twofold upregulation of the expression of IL-15Rα on mDC at day 1 of *Leishmania* infection (Supporting Information Fig. 1). Third, in the viral model the proliferation of the NK cells in the absence of IL-15 (or IFN-α/β) was maintained by the activating NK-cell receptor Ly49H, which is directly targeted by a single, highly stimulatory viral glycoprotein m157, and by IL-12 42,43. Although surface lipophosphoglycan purified from *L. major* was reported to activate human NK cells *in vitro* 44, we obtained no evidence for a direct activation of NK cells by *L. infantum* parasites 10. In fact, recent data suggest that *Leishmania* parasites might even express surface molecules that directly suppress NK-cell proliferation 45.

In conclusion, our analysis of the early NK-cell effector response in visceral leishmaniasis revealed differential functions for the cytokines IL-12, IL-15 and IL-18. In contrast to other models, IL-15 was required for NK-cell proliferation, but not for the IL-12-dependent NK cell IFN-γ production and cytotoxicity. We propose that the NK-cell effector response elicited by different pathogens *in vivo* requires distinct sets of cytokine signals and does not converge on a single or dominant NK-cell activation pathway.

## Materials and methods

### Mice, parasites and infection

Female C57BL/6 mice were obtained from Charles River (Sulzfeld, Germany). IL-12p35^−/−^ 46, IL-18^−/−^ 35 (both on a C57BL/6 background) and B6.SJL-Ptprc^a^Pepc^b^/BoyJ (CD45.1^+^) mice were from the Jackson Laboratories (Bar Harbor, ME, USA). IL-15^−/−^ mice 31 (C57BL/6 background) were purchased from Taconic (Germantown, NY, USA). All mice were housed under specific pathogen-free conditions and used at the age of 6–16 wk.

The origin, propagation and preparation of *L. infantum* promastigotes (strain MHOM/00/98/LUB1) were described before 10.

Mice were injected into the retro-orbital plexus with 300 μL PBS or 1×10^7^ stationary-phase *L. infantum* promastigotes in 300 μL PBS. The animal experiments were approved by the governmental animal welfare committee.

### Generation, purification and stimulation of DC and macrophages

Preparation of splenic mDC, generation of BMMΦ and BM-mDC and cultivation of the cells was performed as described 10,37. For stimulation CpG ODN 1668 (1 μM, Thermo Electron, Ulm, Germany) and *L. infantum* promastigotes (parasite:cell ratio (MOI) indicated in the text) were used.

### Flow cytometry analysis and intracellular cytokine staining

For surface phenotyping and cell sorting the following fluorochrome (FITC-, PE-, PerCP or APC)-labeled or biotinylated mAb were used (all from BD Biosciences, Heidelberg, Germany, unless otherwise stated): anti-CD11b (M1/70), anti-CD11c (HL3), anti-I-A/I-E (M5/114.15.2), anti-F4/80 (CI:A3-1, AbD Serotec, Düsseldorf, Germany), anti-IL-15Rα (BAF551, R&D Systems, Wiesbaden, Germany), anti-NK1.1 (PK136), anti-NKp46 (29A1.4; eBioscience, Hatfield, UK), anti-CD45.1 (A20), anti-CD3 (145–2C11), anti-IL12Rβ1 chain (114) and anti-FasL (MFL3). The specificity of the stainings was verified by the use of isotype control mAb. PI was included at 1 μg/mL in the final wash to detect dead cells.

The intracellular cytokine staining of IFN-γ in splenic NK cells or of IL-12p40/70 in splenic CD11b^int^CD11c^+^ cells was performed after a 4 h *in vitro* restimulation of spleen cells in medium alone or with YAC-1 cells in the presence of brefeldin A 10. In some experiments splenocytes were restimulated *in vitro* with rmIL-18 (10 ng/mL, R&D Systems) for 4 h, washed three times with PBS and stimulated with rmIL-12 (1 ng/mL, R&D Systems) for further 15  h before analysis of intracellular IFN-γ expression in NK cells 10. The intracellular expression of gzmB and perforin in splenic NK cells was measured by anti-gzmB (16G6, eBioscience) and anti-perforin (eBioOMAK, eBioscience) directly *ex vivo*. All FACS analyses were run on a FACS Calibur® (BD Biosciences) using the Cell Quest Pro® software.

### NK-cell proliferation *in vivo*

To investigate cell proliferation *in vivo* the thymidine analog BrdU (1 mg/mouse) was injected i.p. into *L. infantum*-infected, IL-15/sIL-15R-Fc- or poly(I:C)-treated or control mice 22 h p.i., respectively. Two hours later the number of cells that incorporated BrdU into the DNA during proliferation was determined *via* the APC BrdU Flow Kit (BD Biosciences) according to the manufacturer's instructions.

### NK-cell cytotoxicity assay

After determination of the percentage of NK1.1^+^CD3^−^ or NKp46^+^CD3^−^ NK cells within whole spleen cells, the NK-cell cytotoxicity against YAC-1 tumor cells was determined in a standard chromium-51 release assay 10.

### NK-cell transfer and *in vivo* treatments

For cell transfer experiments splenic NK cells of CD45.1^+^ C57BL/6 mice were purified by MACS technology using anti-DX5 MicroBeads (Miltenyi, Bergisch-Gladbach, Germany) and by subsequent MoFlo® sorting gating on NK1.1^+^CD3^−^ cells (purity ≥99%). Purified NK cells were injected i.v. For activation of NK cells *in vivo*, mice received 50 μg poly(I:C) (Invivogen, Toulouse, France) i.p. For neutralization of IL-12 mice were injected with 100 μg rat anti-IL-12p40 mAb (C17.8, eBioscience) or control rat IgG (The Jackson Laboratory) 30 min before i.v. infection with *L. infantum*. As positive control for the NK-cell proliferation assay mice were injected i.p. with pre-complexed 0.5 μg/mouse huIL-15 and 3 μg/mouse sIL-15Rα-Fc (both R&D Systems) as described 34.

### mRNA expression analysis

Total RNA of homogenized tissue or of purified cells was extracted using the TRIZOL reagent (Invitrogen, Karlsruhe, Germany) or the RNeasy Micro Kit (Qiagen, Hilden, Germany) and was reverse transcribed using the High Capacity cDNA Archive Kit (Applied Biosystems, Darmstadt, Germany). To assess the amount of mRNA expression levels we exactly followed our previously described protocol 30. The following assays (Applied Biosystems) were used: murine hypoxanthine guanine phosphoribosyl transferase 1 (mHPRT-1) (Mm00446968_m1), mIFN-γ (Mm00801778_m1), perforin (Mm00812512_m1), gzmB (Mm00442834_m1) and IL-12Rβ2 (Mm00434200_m1).

### Statistical analysis

The non-parametric Mann–Whitney test was performed where indicated and *p* values are given.

## Abbreviations

gzmB: granzyme B
MCMV: mouse CMV
mDC: myeloid DC
p.i.: post infection
