# Supplementary material for: IL-18, but not IL-15, contributes to the IL-12-dependent induction of NK-cell effector functions by Leishmania infantum in vivo
Source: Eur J Immunol. 2010 Mar 8;40(6):1708–17. doi: 10.1002/eji.200939988 (PMC2909391; doi:10.1002/eji.200939988)
Supplement: Supplementary file 1 [file eji0040-1708-SD1.pdf]

European Journal of Immunology

Supporting Information

for

DOI 10.1002/eji.200939988

IL-18, but not IL-15, contributes to the IL-12-dependent induction of NK-cell effector functions by *Leishmania infantum in vivo*

Simone Haeberlein, Heidi Sebald, Christian Bogdan and Ulrike Schleicher

© 2010 WILEY-VCH Verlag GmbH & Co. KGaA, Weinheim

Supplemental Figure S1

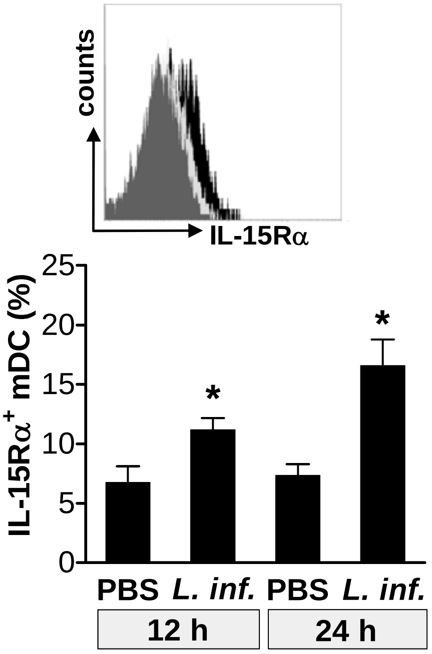

Up-regulation of IL-15Rα on splenic mDC in *L. infantum*-infected mice. C57BL/6 WT or IL-15$^{-/-}$ mice were injected i.v. with 1 x 10^7 *L. infantum* promastigotes or PBS and splenocytes were prepared 12 and 24 h p.i. IL-15Rα surface expression of splenic DC (gated on CD11b^{int}CD11c^{hi} cells) was analysed by FACS after staining with biotinylated polyclonal goat anti-IL-15Rα IgG and streptavidin-APC. As a control cells were stained with biotinylated normal goat IgG and streptavidin-APC. Mean ± SEM of 4 independent experiments with 2 mice per group is given. The histogram overlay represents one example for the IL-15Rα expression 24 h p.i. (black: *L. infantum* infection; light gray: PBS control; dark gray: control Ab staining). Significant differences by Mann-Whitney test between cells from infected and PBS-treated mice (*, $p < 0.05$) are indicated.

Supplemental Figure S2

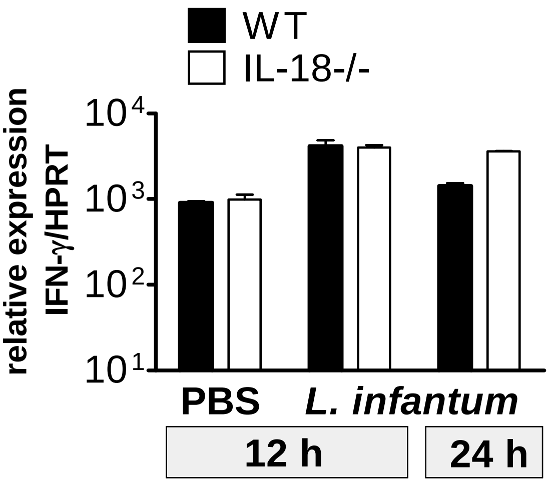

IFN-γ mRNA expression in NK cells of WT and IL-18$^{-/-}$ mice upon infection with *L. infantum*. 1 x 10^7 *L. infantum* promastigotes or PBS were injected i.v. into C57BL/6 WT or IL-18$^{-/-}$ mice. NK1.1$^+$CD3$^-$ NK cells were purified 12 and 24 h after treatment by magnetic bead separation and subsequent MoFlo sorting and total RNA was prepared. The IFN-γ mRNA expression was analysed using quantitative RT-PCR and normalized to the endogenous control gene mHPRT-1. Mean ± SEM of 1 representative out of 3 independent experiments with 2 mice per group is given.

Supplemental Figure S3

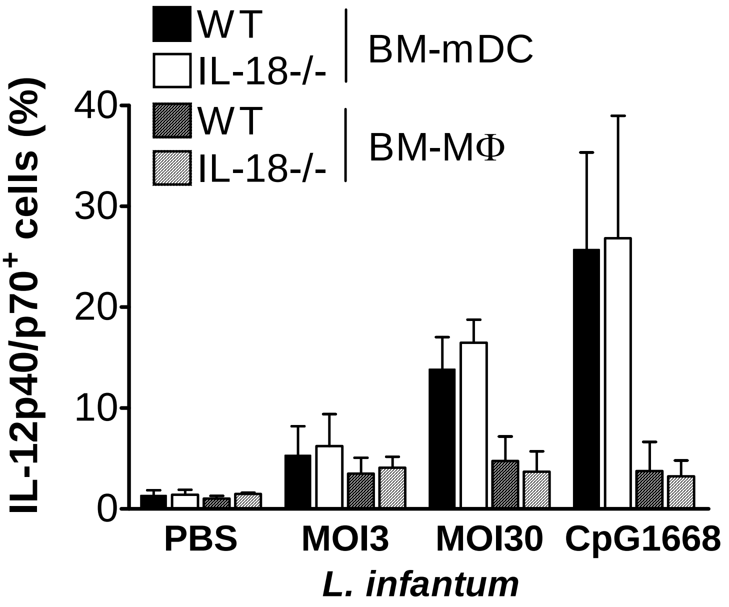

No difference in the IL-12 production by BM-derived mDC and macrophages of WT and IL-18$^{-/-}$ mice after infection with *L. infantum* in vitro. BM-derived mDC or macrophages were stimulated in vitro for 24 h with PBS, *L. infantum* promastigotes (MOI=3 or MOI=30) or CpG1668 (1 µM) and intracellular IL-12p40/p70 protein expression of gated CD11b$^+$CD11c$^+$ mDC or CD11b$^+$F4/80$^+$ macrophages was measured. Mean ± SEM of 3 independent experiments.

Supplemental Figure S4

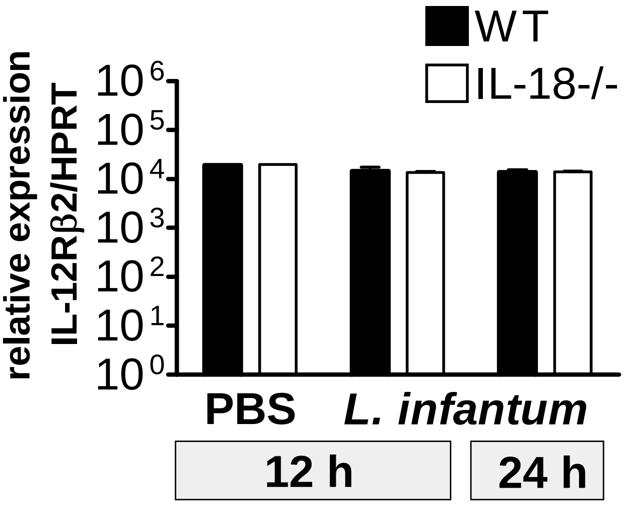

IL-12Rβ2 mRNA expression in NK cells of WT and IL-18$^{-/-}$ mice after infection with *L. infantum*. 1 x 10^7 *L. infantum* promastigotes or PBS were injected i.v. into C57BL/6 WT or IL-18$^{-/-}$ mice and NK cells were purified as described in the legend to Fig. S2. The IL-12Rβ2 mRNA expression was analysed using quantitative RT-PCR and normalized to the endogenous control gene mHPRT-1. Mean ± SEM of 1 representative out of 2 independent experiments with 2 mice per group is given.

Supplemental Figure S5

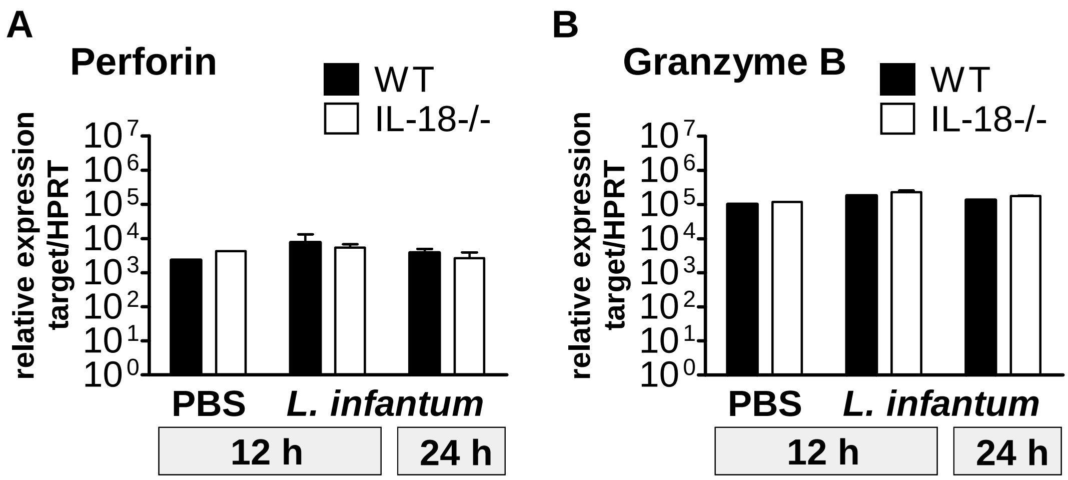

Perforin and granzyme B mRNA expression in NK cells of WT and IL-18$^{-/-}$ mice after infection with *L. infantum*. 1 x 10^7 *L. infantum* promastigote parasites or PBS were injected i.v. into C57BL/6 WT or IL-18$^{-/-}$ mice and NK cells were purified as described in the legend to Fig. S2. The perforin (A) and gzm B (B) mRNA expression were analysed using quantitative RT-PCR and normalized to the endogenous control gene mHPRT-1. Mean ± SEM of 1 representative out of 2 independent experiments with 2 mice per group is given.
